# Effect of immersive virtual reality-based cognitive remediation in patients with mood or psychosis spectrum disorders: study protocol for a randomized, controlled, double-blinded trial

**DOI:** 10.1186/s13063-024-07910-7

**Published:** 2024-01-24

**Authors:** Andreas E. Jespersen, Anders Lumbye, Maj Vinberg, Louise Glenthøj, Merete Nordentoft, Eva E. Wæhrens, Gitte M. Knudsen, Guido Makransky, Kamilla W. Miskowiak

**Affiliations:** 1 Neurocognition and Emotion in Affective Disorders (NEAD) Centre, Psychiatric Centre Copenhagen, Frederiksberg Hospital, DK-2000 Copenhagen, Denmark; 2 Department of Psychology, University of Copenhagen, Copenhagen, Denmark; 3 Wide Angle Media, Copenhagen, Denmark; 4 Department of Clinical Medicine, Faculty of Health and Medical Sciences, University of Copenhagen, Copenhagen, Denmark; 5 Mental Health Services, The Early Multimodular Prevention and Intervention Research Institution (EMPIRI), Mental Health Centre, Northern Zealand, Copenhagen, Denmark; 6 Copenhagen Research Centre for Mental Health-CORE, Mental Health Centre Copenhagen, Copenhagen University Hospital, Copenhagen, Denmark; 7 The Parker Institute, Bispebjerg and Frederiksberg Hospital, University of Copenhagen, Copenhagen, Denmark; 8 Occupational Science, User Perspectives and Community-Based Interventions, Department of Public Health, University of Southern Denmark, Odense, Denmark; 9 Neurobiology Research Unit, University Hospital Rigshospitalet, Copenhagen, Denmark; 10 Virtual Learning Lab, Department of Psychology, University of Copenhagen, Copenhagen, Denmark

**Keywords:** Virtual reality, Cognition, Cognitive remediation, Depression, Bipolar disorder, Schizophrenia, Psychosis

## Abstract

**Background:**

Cognitive impairments are prevalent across mood disorders and psychosis spectrum disorders, but there is a lack of real-life-like cognitive training programmes. Fully immersive virtual reality has the potential to ensure motivating and engaging cognitive training directly relevant to patients’ daily lives. We will examine the effect of a 4-week, intensive virtual reality-based cognitive remediation programme involving daily life challenges on cognition and daily life functioning in patients with mood disorders or psychosis spectrum disorders and explore the neuronal underpinnings of potential treatment efficacy.

**Methods:**

The trial has a randomized, controlled, double-blinded, parallel-group design. We will include 66 symptomatically stable outpatients with mood disorders or psychosis spectrum disorders aged 18–55 years with objective and subjective cognitive impairment. Assessments encompassing a virtual reality test of daily life cognitive skills, neuropsychological testing, measures of daily life functioning, symptom ratings, questionnaires on subjective cognitive complaints, and quality of life are carried out at baseline, after the end of 4 weeks of treatment and at a 3-month follow-up after treatment completion. Functional magnetic resonance imaging scans are performed at baseline and at the end of treatment. The primary outcome is a broad cognitive composite score comprising five subtasks on a novel ecologically valid virtual reality test of daily life cognitive functions. Two complete data sets for 54 patients will provide a power of 80% to detect a clinically relevant between-group difference in the primary outcome. Behavioural data will be analysed using linear mixed models in SPSS, while MRI data will be analysed with the FMRIB Expert Analysis Tool (FEAT). Treatment-related changes in neural activity from baseline to end of treatment will be investigated for the dorsal prefrontal cortex and hippocampus as the regions of interest.

**Discussion:**

The results will provide insight into whether virtual reality-based cognitive remediation has beneficial effects on cognition and functioning in symptomatically stable patients with mood disorders or psychosis spectrum disorders, which can aid future treatment development.

**Trial registration:**

ClinicalTrials.gov NCT06038955. Registered on September 15, 2023.

**Supplementary Information:**

The online version contains supplementary material available at 10.1186/s13063-024-07910-7.

## Background

Moderate-to-severe cognitive impairments are common across mood disorders (MD) and psychosis spectrum disorders (PSD) [1–3] with studies suggesting a prevalence of approximately 50% in remitted patients with major depressive disorder [4], 50–70% in remitted patients with bipolar disorder [5, 6], and up to 80% in patients with schizophrenia [7, 8]. Importantly, the impairments often persist during asymptomatic phases of illness [2, 4, 5] and are directly associated with poorer prognosis, increased functional disability, and reduced work capacity [9–12], with the latter comprising the largest socioeconomic burden of the disorders [13, 14]. The impact of cognitive impairments on functional ability highlights the need for novel therapies to not only enhance cognition but also improve daily life functioning [6, 15, 16]. Cognitive remediation (CR) is an umbrella term for therapeutic interventions that focus on ameliorating cognitive impairments through cognitive training, cognitive rehabilitation, and cognitive stimulation [16–18]. CR with a focus on cognitive training is a particularly promising approach with well-established effects on cognitive and functional outcomes in PSD [19, 20] and, more recently, findings of moderate benefits in cognitive function in MD [18, 21].

WHO’s International Classification of Functioning, Disability and Health (ICF) [22] provides a useful framework for distinguishing between cognitive *functions* such as memory or attention (i.e. the ICF level of body functions) and cognitive *skills* necessary for carrying out daily life tasks (i.e. the ICF level of activity/participation). Cognitive training in CR interventions typically targets both of these levels, aiming to normalize cognitive *functions* through ‘drill-and-practice’-based approaches, such as computer-based exercises, and improving cognitive *skills* by teaching patients to apply the cognitive gains in daily life and practicing compensatory strategies that aid daily life activities [16, 23, 24]. A strategy-based approach focusing on training cognitive skills has been demonstrated to be particularly important for increasing the transfer effects of cognitive improvement to functional outcomes in patients’ daily lives [16, 24]. Nevertheless, most CR interventions still find *limited transfer* of acquired cognitive skills to daily life functioning, which puts into question the clinical impact of the interventions [18, 20, 24]. Several challenges may contribute to the limited transfer effect. First, cognitive training rarely involves the possibility of *directly* training cognitive skills within actual challenging real-life situations, and it seems insufficient to merely discuss with patients in the therapy sessions how strategies can be applied in daily life [21, 24, 25]. Indeed, meta-analytic evidence from CR trials in PSD suggests that transfer is greater when CR is combined with more structured psychosocial rehabilitation such as vocational training [26]. Second, treatment effects in CR programmes are often also limited by difficulty in sufficiently motivating participants to complete the cognitive training despite therapist encouragement and support [27]. It has been suggested that participants may lose interest in the training, perhaps because the relatively abstract computer exercises or strategy discussions do not directly relate to their daily life cognitive challenges and therefore do not seem directly relevant [24, 28]. As such, some programmes suffer from poor treatment adherence, and attrition rates as high as 50% have been reported [21, 27, 29]. Together, these challenges emphasize the need for developing and integrating more ecologically valid and engaging techniques in CR interventions to aid transfer and treatment adherence [17, 21, 24].

Virtual reality (VR) platforms have the potential to overcome these challenges because of their highly engaging and real-life-like format. In VR, users can be immersed in naturalistic and multimodal simulations of cognitively challenging daily life scenarios that are quite like situations that patients may encounter in their daily lives [30–33]. VR thereby provides an ecologically valid method for directly practicing the cognitive strategies that are discussed in therapy in a real-life-like safe setting. Notably, studies of VR and learning have found that fully immersive VR, such as a head mounted display (HMD), has a better effect on learning performance than non-immersive learning approaches and improves knowledge transfer to solve real-world tasks [34–36]. It therefore seems reasonable to assume that VR can enhance bridging between cognitive training and daily life cognitive skills in CR interventions and facilitate greater transfer effects [33]. VR simulations using HMDs also induce an increased feeling of being ‘present’ in the virtual environment by effectively shutting out the physical reality [37, 38]. In educational contexts, an increased feeling of ‘presence’ has been shown to influence engagement in VR users by boosting interest and motivation [39]. Similar effects in CR programmes using gamified VR platforms could help instil greater motivation in patients and increase treatment adherence. A recent systematic review found initial promising evidence for the use of fully immersive VR-based cognitive training in neuropsychiatric disorders, particularly if training is relevant for daily life challenges [40]. However, cognitive intervention studies in MD and PSD using VR are still scarce and suffer from various methodological challenges, including a lack of active control groups, small sample sizes, and poor descriptions of intervention components [40–43]. To address this gap, our research group developed a fully immersive VR prototype training scenario in a virtual kitchen environment with input from individuals with MD or PSD in line with recommendations to include expert-by-experience knowledge [44, 45]. We then conducted a randomized, controlled proof-of-concept study in 40 participants with MD or PSD in which the active group trained various cognitive strategies in the VR protype training scenario [44]. This study demonstrated that short-term (1 week), intensive VR-based cognition training had the potential to improve some aspects of cognitive performance [44]. Participants also rated the VR prototype scenario as being a fun, engaging, and safe training environment that felt relevant for their daily lives [44].

A general methodological challenge in developing treatments targeting cognition is the lack of insight into the neurocircuitry targets of pro-cognitive interventions [46]. Therefore, recent recommendations by the International Society for Bipolar Disorder (ISBD) Targeting Cognition Task Force underscore the need for implementation of neuroimaging in cognitive intervention trials to increase insight into brain-based mechanisms for treatment effects [47]. A recent systematic review of studies using functional MRI found that mood disorders (MD) are consistently associated with abnormal (primarily hypo-) activity in the dorsal prefrontal cortex (dPFC) and hyperactivity in the default mode network (DMN) during working memory and strategic encoding [48]. Hypoactivity in the dPFC is also a consistent finding in patients with PSD [49]. There is growing evidence for treatment-related changes in dPFC activity following CR interventions across MD and PSD [50–52]. In keeping with this, a meta-analysis of changes in brain activity following CR in PSD found increased activity in the dPFC to be the most robust brain-based marker for cognitive improvement [50]. In our previous study of action-based cognitive remediation (ABCR) in MD, we found early treatment-related activity in the dPFC during working memory, which predicted improved executive functions after 10 weeks of treatment [53]. Together, these findings suggest that changes in the prefrontal cortex may be a potential biomarker for the effect of treatment across PSD and MD.

### Aims and hypothesis

This study aims to assess the effect of a 4-week, intensive VR-based cognitive remediation programme involving daily life challenges on cognition in symptomatically stable outpatients with MD or PSD. Additionally, we will investigate whether any treatment-related cognitive improvement translates into improved daily life functioning and is accompanied by a change in dorsal prefrontal activity. We hypothesize that VR-based cognitive remediation vs. a VR control treatment has a beneficial effect on cognition after 4-week treatment completion (primary outcome assessement time) measured with a novel ecologically valid VR test of daily life cognitive skills (primary outcome measure), a verbal learning and memory composite score based on a traditional neuropsychological test, and a performance-based measure of daily life functioning (secondary outcome measures). For exploratory purposes, we will examine neuronal underpinnings of treatment effects and effects on additional measures of cognition, functioning, and self-rating scales (tertiary outcomes).

## Methods

### Study design and participants

**Fig. 1:**
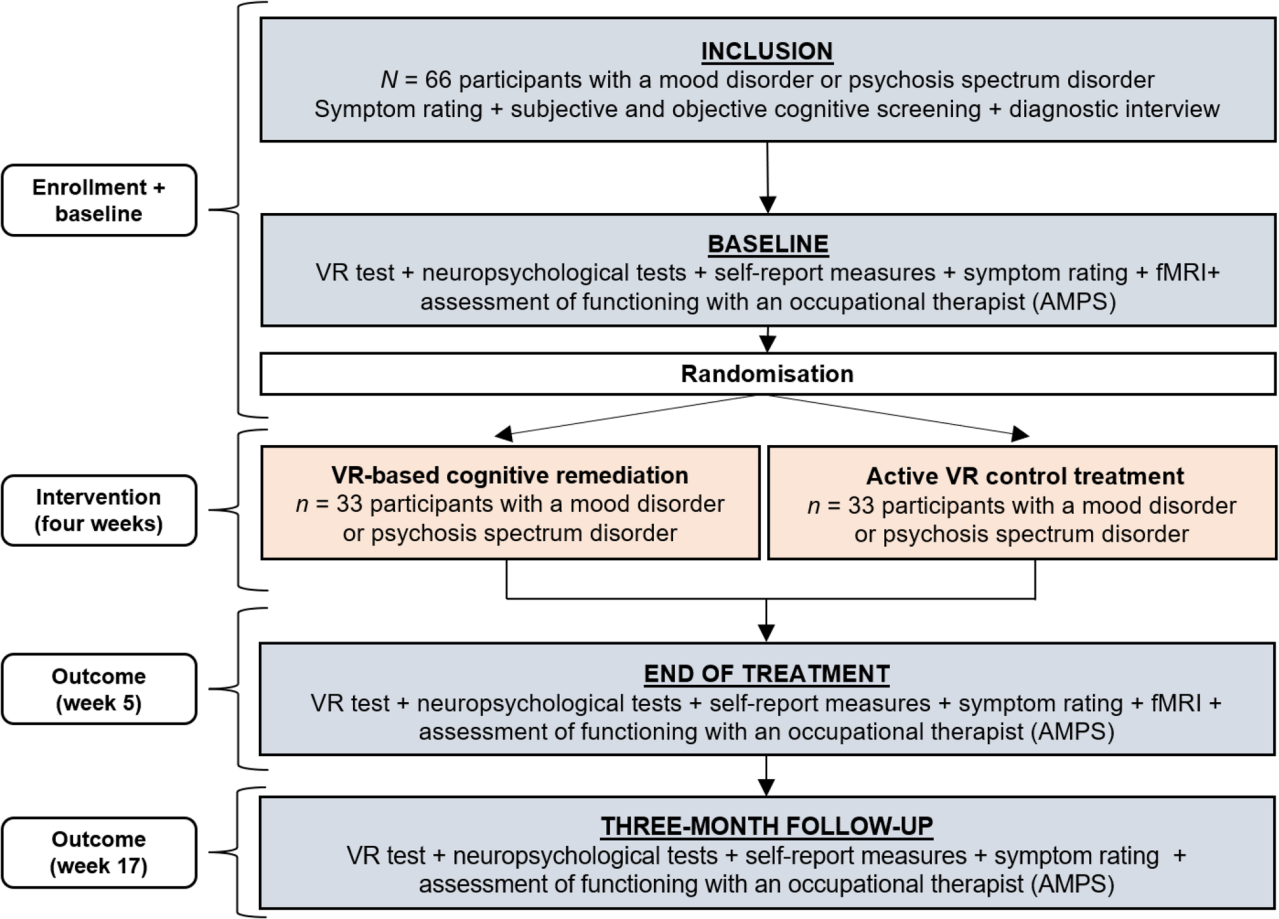
Flow chart of the study design

See Fig. 1 for a flow diagram of the trial. The project has a randomized, controlled, double-blinded, parallel-group design. We will include 66 trial participants with MD (unipolar — or bipolar disorder) in full or partial remission at the time of inclusion (score ≤ 14 on the Hamilton Depression Rating Scale [54] and Young Mania Rating Scale [55], respectively) or PSD (F20 spectrum) that have been assessed to be relatively symptom stable by their treating clinician upon referral to the study. Recruitment will be carried out through the Copenhagen Affective Disorder Clinic, the outpatient, early intervention clinics for psychotic disorders (OPUS), other mental health centres in the Capital Region of Denmark, and through advertisements on relevant websites.

Eligible participants must be between 18 and 55 years of age, be fluent in Danish, and have an ICD-10 diagnosis of unipolar disorder (F32–F33), manic episode or bipolar disorder (F30–F31), or a psychosis spectrum disorder (F20–F29) confirmed using the Schedules for Clinical Assessment in Neuropsychiatry (SCAN) interview [56] and present with both *objective* and *subjective* cognitive impairment in line with expert recommendations [47]. *Objective* cognitive impairment is assessed with the Screen for Cognitive Impairment in Psychiatry-Danish version (SCIP-D) [57] and is defined as a SCIP total score ≥ 0.5 standard deviation (SD) below the expected total score based on age, education, and gender or as a score ≥ 0.5 SD below the expected score on a minimum of two out of the five subtests (verbal learning test—immediate, working memory test, verbal fluency test, verbal learning, test—delayed, and processing speed test) [58]. *Subjective* cognitive impairment is assessed with the Cognitive Complaints in Bipolar Disorder Rating Scale (COBRA) [59] and is defined as a score ≥ 14 [59, 60]. To ensure participant motivation, *subjective* cognitive impairment is also assessed with the Cognitive Difficulties in Everyday Life (CODEL) questionnaire, which has been developed in house to specifically assess cognitive complaints within the challenging daily life situations that are targeted by the VR intervention (see details under ‘intervention’). On the CODEL, *subjective* impairment is defined as a score ≥ 7 in a minimum of two of the four subdomains on the questionnaire (i.e. cooking, shopping, remembering verbal information, and planning). Exclusion criteria are current drug or substance abuse, a daily use of benzodiazepines > 22.5-mg oxazepam or > 7.5-mg diazepam (cut-offs for doses with limited cognitive side effects), comorbid neurological disorder or previous serious head trauma, dyslexia, pregnancy, claustrophobia, having a pacemaker or other metal implants inside the body, and electroconvulsive therapy in the 3 months prior to inclusion. All participants must provide written informed consent. The SPIRIT reporting guidelines were used for the current study [61]. See Appendix A for a completed SPIRIT checklist.

### Procedure

Upon their first visit at the Copenhagen Affective Disorder Research Centre (CADIC) or Department of Psychology, University of Copenhagen, participants are informed about the project and provide written informed consent, after which they undergo an eligibility assessment. The first author is responsible for recruitment and conducting the first visit during which the informed consent is obtained. After inclusion, the baseline assessments are scheduled and completed over 2 days, 1 to 5 days apart for practical reasons and to avoid attrition.

On day 1, the participant is assessed with a novel VR cognition test of daily life cognitive skills and a comprehensive neuropsychological test battery (see details under ‘Outcome measures’). Participants also complete questionnaires concerning subjective cognitive complaints, quality of life, and general acceptance of VR technology. Daily life functioning is assessed using a clinician-rated interview and a performance-based assessment. Participants with MD undergo mood ratings with the HDRS-17 and YMRS. For participants with PSD, positive symptoms are assessed using the Scale for the Assessment of Positive Symptoms (SAPS) [62], and negative symptoms are assessed using the Brief Negative Symptom Scale (BNSS) [63]. Finally, self-reported sleep quantity and quality in the past 3 days are recorded. At the Copenhagen University Hospital, Rigshospitalet, participants then undergo a functional magnetic resonance imaging (fMRI) scan encompassing a spatial working memory N-back task, a word encoding paradigm in which participants must encode and subsequently recognize words of typical household items, a resting state, and a structural scan.

On day 2, participants’ ability to perform daily life tasks is assessed with the Assessment of Motor and Process Skills (AMPS) [55] by an AMPS-certified occupational therapist in a standardized test kitchen.

The VR cognition test, neuropsychological assessments, questionnaires, clinical symptom ratings, assessments of functioning (including AMPS), and fMRI scan are repeated within 2 weeks after treatment completion (primary outcome assessment time). All assessments except for the fMRI scan are repeated 3 months after treatment completion to assess durability.

Participants are requested to avoid significant changes to the dose and type of any medication prior to or during the study. Any changes in the type or dose of medication will be recorded at treatment completion and at the 3-month follow-up. Patients are not excluded from the project if their symptoms worsen unless they are incapable of engaging in the treatment or voluntarily decide to drop out.

### Randomization and blinding

The study has a double-blinded design. Neither the participant nor the outcome assessors will know whether the participant is receiving VR-based cognitive remediation or VR control treatment (details below). At inclusion, participants are informed that they will be randomized to one of two types of VR training programmes which both involve weekly sessions with a therapist. To uphold the blinding, participants are not provided with any further details regarding the VR-based cognitive remediation and control treatment until after randomization. Participants are randomized upon full completion of the baseline assessment with allocation being carried out by the first author using the automated randomization module in the online Research Electronic Data Capture (REDcap) system based on an uploaded blocked randomization list created online using Sealed Envelope Ltd. 2022 [64]. Participants will be randomized using a 1:1 allocation in blocks of varying size from 4-8, and we will stratify randomization with age (< or ≥ 35 years) and diagnosis (MD vs. PSD) to ensure equal distribution in the two groups. To avoid any predictability in the randomization process, the list detailing the randomized blocking sequences is kept inaccessible to the first author who enroll and randomize the participants. The participants will be instructed not to disclose any information concerning their treatment during outcome assessments and under no circumstances will the allocation be revealed to the outcome assessors. Outcome assessors will be asked to note whether the blinding was compromised during the assessment. The first author who undertakes the statistical analyses will be blinded with respect to group assignments when conducting the primary and secondary outcome analyses.

### Study setting

The CAVIR trial will be conducted at CADIC, Psychiatric Center Copenhagen, Frederiksberg Hospital, and at the Department of Psychology, Copenhagen University, Denmark. MR scans are conducted at the Copenhagen University Hospital, Rigshospitalet, Copenhagen, Denmark.

### Intervention: virtual reality-based cognitive remediation

The VR-based cognitive remediation intervention is primarily strategy based and comprises individual psychoeducation combined with training of cognitive skills in VR daily life scenarios that helps participants practice and transfer learned cognitive strategies. The scenarios and cognitive strategies have all been chosen and developed based on feedback from participants in our proof-of-concept study [44], our previous group-based CR intervention [25], and with input from students with MD or PSD attending the School for Recovery at Psychiatric Center Copenhagen [65] who participated in a focus-group interview. These steps were taken to ensure the highest possible relevance of the intervention in line with recommendations to include expert-by-experience knowledge [45]. Additionally, the scenarios were developed in collaboration with clinicians at the Copenhagen Affective Disorder Clinic. All scenarios were pilot tested for feasibility in a minimum of three test participants and optimized further based on their feedback.

**Fig. 2:**
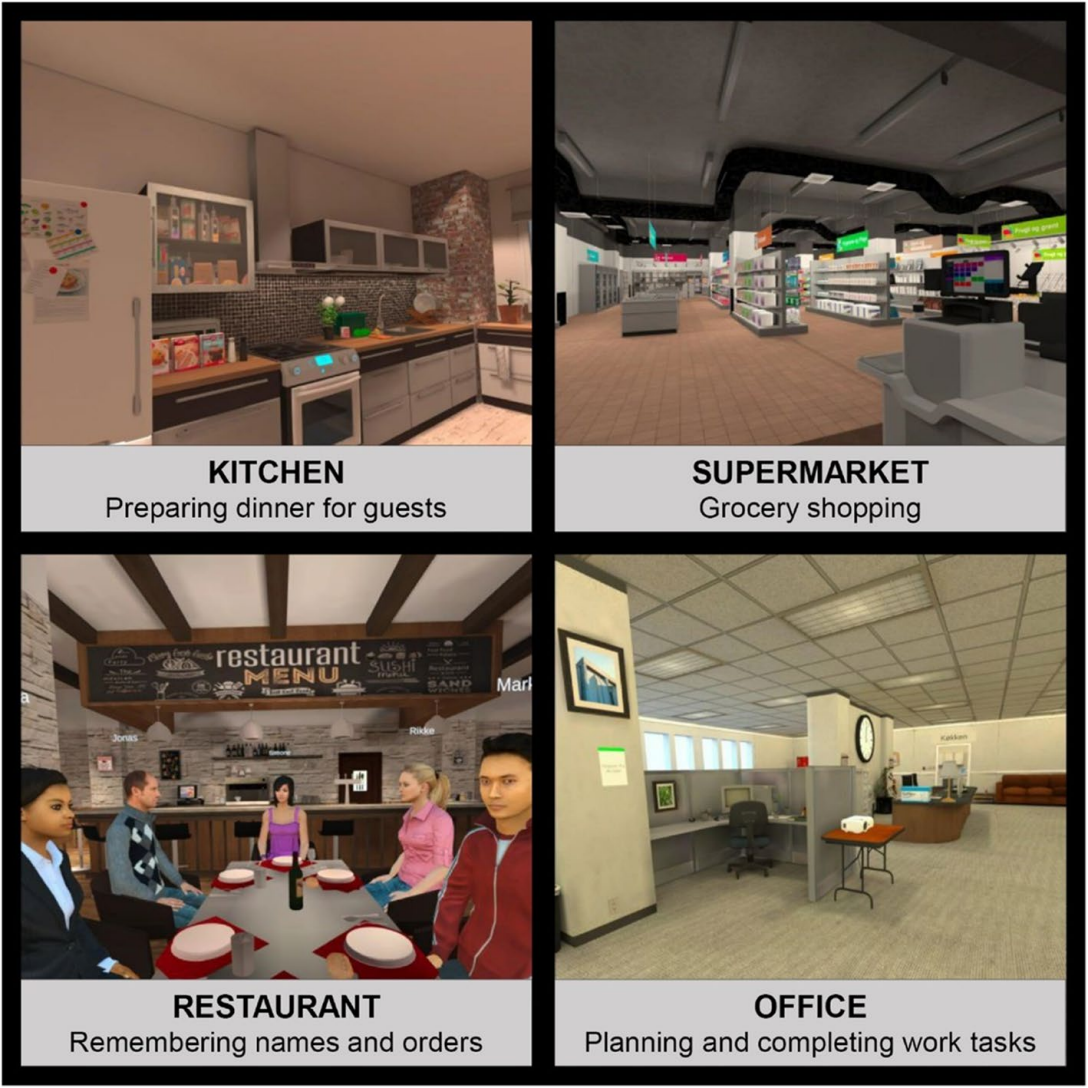
Virtual reality training environments

The short-term, intensive programme has a duration of 4 weeks and involves two weekly 2-h training and bridging sessions with a therapist accompanied by additional between-session VR training at home and homework assignments consisting of cognitively challenging daily life tasks. The main component of the intervention is the fully immersive VR training platform in 360° Oculus Quest 2 software [66]. The platform includes four daily life scenarios in which patients have consistently reported that they often experience cognitive difficulties: 1) a kitchen scenario focusing on planning and cooking a meal, 2) a supermarket scenario focusing on grocery shopping, 3) a restaurant scenario focusing on remembering names and personal information, and 4) an office scenario focusing on planning, initiating, and completing work assignments (see Fig. 2). Each scenario has a duration of 15–30 min per level and comprises multiple subtasks targeting different types of daily life cognitive skills (e.g. remembering items from a shopping list). All scenarios are designed based on an adaptive learning methodology to ensure optimal learning and motivation [21, 67]. Specifically, there are between three to six levels of varying difficulty in the scenarios, and participants require an 85% success rate to advance to a more challenging level [67]. Participants are introduced to a new VR scenario each week, starting with the kitchen scenario (low VR skill level requirement) and finishing with the office scenario (high VR skill level requirement). The scenarios all include an introductory module and CR strategies embedded directly in the VR environment that are presented to the participant before they complete the tasks.

The virtual reality training is supported by a psychoeducational programme that focuses on the application of learned cognitive strategies in daily life. The programme is designed to match the content of the weekly VR training scenario and covers the following themes: meta-cognition and seeking out cognitive challenges (week 1), strategic learning and memory strategies (week 2), verbal memory and sustaining attention (week 3), and organizing and prioritizing (week 4). The first session also introduces the structure of the treatment and focuses on individual goal setting, including identification of cognitive strengths and weaknesses based on the screening carried out during the eligibility assessment. Each session consists of a short presentation of the theme of the day followed by training in VR (2 × 30 min) and a discussion of how the VR training is related to the individual experiences and goals of the participant. Each session ends with identifying cognitively challenging tasks for the participant to carry out between sessions (e.g. cooking a meal, going shopping without a list, remembering conversation, reading, or organizing and completing tasks at home). Participants then take a head-mounted display (HMD) home to conduct between-sessions VR training (2 × 30 min of home training per week). Treatment completion is defined as 80% attendance corresponding to full completion of six out of eight in-person training sessions. When a participant is unable to attend a scheduled session, the therapist will help the participant catch up on the session’s content through additional at-home training or by extending the subsequent treatment session. The VR training at home is recorded for subsequent evaluation of feasibility. At the final session, participants return the HMD, and a discussion takes place on whether and how they benefited from the intervention, along with encouraging them to keep practicing learned skills. They also receive a leaflet with information from the training sessions.

### Virtual reality control treatment

Participants in the control group attend a weekly 2-h session with a therapist during which they complete different VR games that are available through the Oculus Quest games store [68]. The chosen games involve no direct training of cognitive skills, such as planning skills or strategic learning, but merely involve simple reaction time and interaction with an entertaining environment that is meaningful to the participant. At the session, participants also complete alternative versions of the same VR training scenarios as the training group (i.e. kitchen, supermarket, restaurant, and office) in which the training elements (i.e. strategies, adaptive difficulty level, and feedback) have been removed. These control sessions are conducted for three reasons: (i) to assess whether a potential effect on the VR cognition test (primary outcome measure; see details below) reflects a therapeutic effect rather than simple perceptual habituation to VR environments (i.e. becoming better at using and interacting with VR), (ii) to control for the nonspecific effects of meeting weekly with a therapist, and (iii) to blind study participants in both groups so that all participants have the impression of training their cognitive functions and daily life cognitive skills in VR. To ensure engagement in the control group, participants are asked at the end of each session to complete questionnaires evaluating their experiences with the VR games and scenarios.

### Outcome measures

#### Virtual reality-based assessment of daily life cognitive skills

The effect of the intervention is assessed with a novel VR test of daily life cognitive skills, the Cognition Assessment in Virtual Reality (CAVIR) test [69]. The CAVIR test is an engaging, immersive, and self-administered 360° VR test in a kitchen, where the participant’s cognitive skills related to planning and preparing a meal are assessed [69]. The test has proven high validity, sensitivity, and feasibility for cognitive assessment in MD and PSD [69] and can be considered a more ecologically valid alternative to neuropsychological assessment, as it enables insight into patients’ daily life cognitive skills (i.e. the ICF level of activity/participation) which is the primary target of the intervention. The test has a duration of 15 min and involves five subtasks probing different cognitive skills and underlying cognitive functions: 1) memorizing ingredients from a list (verbal memory), 2) planning the order in which to complete subtasks involved in cooking a meal (executive functions), 3) placing as many correct ingredients as possible in a pot within a time frame (processing speed), 4) memorizing the location of cutlery and flatware in the kitchen cupboards and drawers (working memory), and 5) repeatedly checking the food in the oven in response to a specific combination of cues while ignoring irrelevant stimuli (sustained attention) (for details, see supplementary materials in Appendix B). Importantly, the CAVIR test differs in content from the kitchen scenarios used in the intervention and control treatment, although they all comprise similar subtasks in a VR kitchen environment. Specific differences between the test and training scenarios include the kitchen design and setup (e.g. different interior and placement of items) and test stimuli as different meals are prepared (e.g. different ingredients and visual/auditive cues). The CAVIR test is available in two parallel versions, and participants are assessed with different test versions across assessment points in a counterbalanced design to minimize contamination due to test familiarity.

#### Neuropsychological assessment of cognitive functions

The VR-based cognitive remediation primarily targets daily life cognitive skills, but we also expect that the drill-and-practice component of the intervention will improve underlying cognitive functions (i.e. the ICF level of body functions). Cognitive functions are therefore assessed by a trained research assistant using a broad test battery comprising the following traditional neuropsychological tests: the One Touch Stocking of Cambridge (OTS), the Spatial Working Memory test (SWM) and the Rapid Visual Information Processing (RVP) from CANTAB (Cambridge Cognition Ltd.), the Rey Auditory Verbal Learning Test (RAVLT) [70], WAIS-III Letter-Number Sequencing [71], RBANS Coding and Digit Span [72], verbal fluency [73], and Trail Making Test A and B [74]. Premorbid verbal IQ is assessed with the Danish Adult Reading Test [75].

#### Assessment of daily life functioning

The potential transfer effect of the intervention to daily life functioning will be assessed with the Assessment of Motor and Process Skills (AMPS) [76]. The AMPS is a standardized observation-based occupational therapy evaluation developed to describe and measure a person’s ability to perform activities of daily living (ADL) tasks. When administering the AMPS, the participant is first interviewed about their ADL task performance, to identify standardized ADL tasks that are both relevant to the person and represent a sufficient challenge to the person (from simple to more complex cooking, cleaning, and other household tasks). Next, the participant chooses and performs at least two of the ADL tasks while being observed by an AMPS calibrated occupational therapist. During an AMPS evaluation, two domains of performance are evaluated: ADL motor skills (16 items) and ADL process skills (20 items) (for further details, see Appendix B). Several studies support that the AMPS ADL motor and process ability measures are reliable and valid among persons with psychiatric illness and cognitive impairment [77]. A clinically relevant difference has been determined as ≥ 0.3 logit on the ADL motor or process ability scales [76]. Functioning is also assessed with the UCSD Performance-Based Skills Assessment B (UPSA-B) [78, 79] and the clinician-rated interview Functional Assessment Short Test (FAST) [80].

#### Self-report measures

Subjective cognitive impairment is assessed using the COBRA [59] and CODEL. Quality of life and perceived competence are assessed with the World Health Organization’s Quality-of-Life Assessment (WHOQOL-BREF) [81] and a modified version of the Perceived Competence Scale (PCS) [82], respectively. The user experience in the VR environments is assessed using the following questionnaires: the Presence Questionnaire (PQ) [83, 84], the Multimodal Presence Scale for virtual reality environments (MPS), [85] a modified version of the Technological Acceptance Model (TAM) questionnaire [86], the Virtual Reality Simulation Sickness questionnaire (VRSQ) [87], and a scale comprising items assessing usability and enjoyment composed by our collaborators at the Virtual Learning Lab at the University of Copenhagen. Finally, a user feedback survey has been created by our group to assess participants’ experience and satisfaction with the intervention programme and control treatment.

#### Functional MRI paradigms

During the functional MRI scans, we will administer a spatial N-back working memory task from our previous studies [46, 53] and a word encoding paradigm in which participants must encode and subsequently recognize words of typical household items. The word encoding paradigm was developed in house to capture strategic encoding and memory and exists in two parallel versions that are administered in a counterbalanced design to minimize learning effects.

#### Primary, secondary and tertiary outcomes

**Fig. 3:**
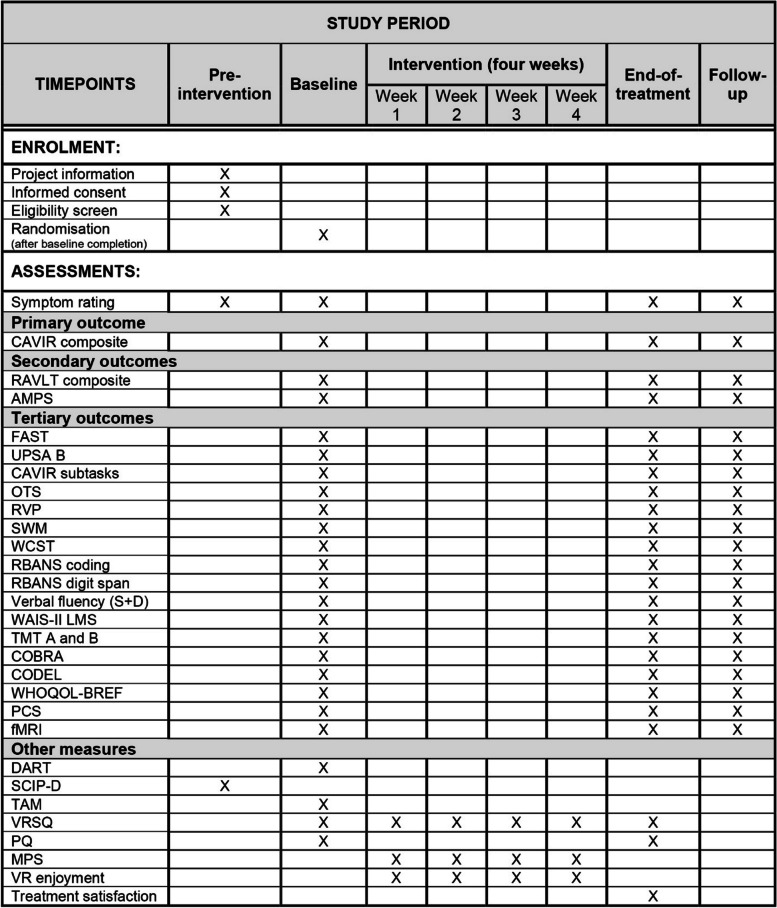
Schedule of enrolment, interventions and assessments

For an overview of the frequency and timing of the outcome measures, see Fig. 3. The primary outcome measure is a broad cognitive composite score comprising all five subtasks on the CAVIR test measuring daily life cognitive skills [69]. The secondary key cognitive outcome measure is a domain composite of ‘verbal learning and memory’ comprising the following RAVLT [70] subtests: RAVLT total recall lists I-V, RAVLT immediate recall, and RAVLT delayed recall as we hypothesize that the intervention will specifically improve verbal learning and memory. The secondary outcome measure of daily life functioning is the AMPS [76]. The tertiary (explorative) outcome measures include subtasks on the CAVIR test and individual cognitive domains based on the traditional neuropsychological test battery. Tertiary outcomes of functional capacity, quality of life, and subjective cognitive impairment are UPSA-B [78], FAST [80], WHOQOL-BREF [81], COBRA [59], and CODEL, respectively. The cognitive and functional outcomes are in line with recommendations for cognition trials from the Targeting Cognition Task Force of the ISBD [47]. Specifically, the recommendations are to include a cognitive composite score as the primary outcome, a single key cognitive measure as the secondary outcome combined with an assessment of daily life functioning to investigate possible transfer effects and multiple individual cognition measures as tertiary outcomes [47].

### Statistical considerations

#### Primary, secondary and tertiary outcome analyses

To investigate the effect on cognition of VR-based cognitive remediation, data will be analysed using linear mixed models for both primary and secondary outcome measures using the Statistical Package for the Social Sciences (SPSS) version 28. For the primary outcome measure, the dependent variable will be the CAVIR cognitive composite score, which is derived by averaging the five z-transformed subtest scores [69]. Data for neuropsychological tests, level of functioning, subjective cognition, and quality of life will also be analysed using mixed models. Intention-to-treat (ITT) analyses will be performed for missing data using a mixed models approach without any ad hoc imputation [88]. Data will be analysed for every randomized participant for all assessments. Interim analyses will not be conducted given the study’s nature and scale.

#### Functional MRI analyses

fMRI data will be preprocessed and analysed with the FMRIB Expert Analysis Tool (FEAT; latest version available at trial completion) part of FMRIB’s Software Library (FSL; www.fmrib.ox.ac.uk/fsl). fMRI data from the N-back working memory task and the supermarket strategic encoding task will be analysed using a region-of-interest analysis to assess differences between the VR-based cognitive remediation and control groups in neural activity in the dorsal PFC (dPFC) after completing 4 weeks of treatment (adjusting for any difference in neural activity at baseline). We will also conduct volume-of-interest analyses of the dPFC for the N-back task and for both the dPFC and hippocampi for the word encoding task to investigate our hypothesis. Finally, exploratory whole-brain analyses will be conducted to investigate any effects in other brain regions.

#### Sample size and power calculation

The power calculation was performed with the software program G*Power 3.1.9.7 [89]. In the proof of concept of our VR prototype training scenario, we observed a 0.6-point (z-scores) greater improvement in the CAVIR cognitive composite score (primary outcome measure in the present trial) in the VR training compared with a treatment as usual (TAU) group with a 0.9 SD of the change [44]. The power calculation assumed normally distributed data and used a two-tailed sample *t*-test. Based on these parameters, the power calculation revealed that 27 participants per treatment arm are needed to achieve > 80% power for detection of a similar cognitive improvement of at least 0.6 SD (corresponding to a moderate effect size) in the CAVIR training group vs. the control group at an alpha level of 0.05. To accommodate for an approximately 20% drop-out rate from baseline to treatment completion, we will recruit 66 participants (33 participants per intervention arm) to obtain complete data for a minimum of 54 participants (27 participants per arm).

#### Data management and monitoring

All personal information will be obtained at the eligibility assessment or from patient records in cases where participants are unable to provide the needed information. Pseudo-anonymized data from the neuropsychological tests, virtual reality test, questionnaires, interviews, and functional assessment will be registered in the REDcap database, which meets requirements from the Danish Data Protection Agency. Data quality is ensured by score range restrictions on values for all outcomes, and all data for the primary and secondary outcome measures will be double-checked by the first author. Signed consent forms as well as a list that matches participant ID numbers with personal information are kept separate from pseudoanonymized data. The list matching participants’ personal information with their ID number will be deleted and consent forms maculated 10 years after study completion. At this point, all data will be completely anonymized. All trial authors will have access to the final trial data set. If a participant is excluded from or withdraws from the study, the reason for exclusion will be documented in REDcap, along with information regarding any adverse events.

#### Participant retention

All participants will be offered feedback on the results of their neuropsychological assessments after completing the 3-month follow-up assessment. They also receive a gift card of 600 Danish crowns, and their travel expenses for public transportation will be reimbursed. For ethical reasons and to ensure motivation, participants randomized to the control treatment will be offered to try an adapted, shorter version of the VR-based cognitive remediation programme following their 3-month follow-up assessment. Specifically, this adapted programme will involve two sessions (60 min) with one training scenario of their choice and will correspond to the intervention from our proof-of-concept study, which participants found meaningful and beneficial [44].

## Discussion

The present study investigates the effect of VR-based cognitive remediation on cognition and functioning in symptomatically stable patients with MD or PSD. The trial thereby addresses a crucial need for exploring more real-life-like cognitive training techniques that can aid the *transfer* of acquired cognitive skills to daily life functioning in CR interventions [16, 17, 24]. The study also investigates neural activity changes associated with improvements in cognition and thereby contributes to the broader objective of identifying a potential biomarker for the effect of pro-cognitive treatments in these patient groups.

The inclusion of both an ecologically valid VR test of daily life cognitive skills and a recommended measure of activities of daily living (ADL) ability will provide insight into whether the VR-based intervention benefits cognitive skills and functioning in daily life, which is the goal for our participants. We hypothesize that VR may help facilitate greater transfer effects by optimizing the possibility for bridging therapy and cognitively challenging activities of daily life. In our proof-of-concept study, participants noted that the VR scenario felt like a safe and fun platform for practicing daily life cognitive skills and strategies [44]. These initial findings suggest that the VR training scenarios could provide a steppingstone for patients to engage or reengage in cognitively challenging and stimulating situations that may otherwise be avoided when they are perceived as too difficult. In keeping with this, VR may also help destigmatize the cognitive difficulties that a large proportion of patients experience despite relative symptom stability [90, 91]. Indeed, the possibility of identifying difficulties and formulating strategies using a gamified, fun, and interesting frontier technology may help to reduce the adverse effects of stigma on self-efficacy and treatment engagement in people with mental illness and cognitive impairment [92, 93].

In our proof-of-concept study, we found that more severe objective cognitive impairments pre-treatment were associated with cognitive improvement in the intervention group [44], which corresponds to previous findings that baseline cognition predicts response to pro-cognitive treatment [47]. In keeping with recommendation by the ISBD Targeting Cognition Task Force [47], eligible participants must therefore present with objective cognitive impairment on the SCIP to ensure that the sample is enriched for cognitive impairment. However, we recognize that neuropsychological tests, such as the SCIP, do not fully capture cognitive impairment in daily life [94]. Therefore, we apply relatively mild criteria for objective baseline impairments on the SCIP (as described in detail under ‘Study design and participants’) to ensure the presence of minimum subtle cognitive impairment while reducing the risk of recruitment challenges from rejecting candidates that may benefit from the treatment. To ensure motivation and treatment relevance, we also include clinically relevant subjective difficulties on the COBRA and CODEL as additional eligibility criteria in the pre-screening. In our proof-of-concept study, we recruited 40 patients with MD or PSD from the Psychiatric Centre Copenhagen [44]. Based on this pilot trial and our collaboration with several recruitment channels including the Copenhagen Affective Disorder Clinic, the outpatient, early intervention clinics for psychotic disorders (OPUS), and other mental health centres in the Capital Region of Denmark, we consider recruitment of 66 patients over 26 months to be feasible.

There are no known direct risks associated with study participation. Every participant is under the coverage of the public insurance institution, the Patient Compensation Association. Some people may experience discomfort (e.g. dizziness) when exposed to immersive VR [95]. However, in our previous studies using similar VR scenarios, we found that participants experienced a low degree of ‘simulation sickness’ during cognitive testing [69] and training in VR [44]. Nevertheless, all exposure to VR will be closely monitored by the therapist. The VR training scenarios have all been designed with a difficulty level that adapts to the individual performance (85% success rate) to maintain motivation without exceeding one’s capacity [67]. The traditional neuropsychological tests, functional assessment, and fMRI tasks are also challenging and may be overwhelming to some participants. We will acknowledge such feelings and seek to reduce them by offering breaks and underscoring that the tasks are generally very challenging, and that their effort is valuable. Overall, the results of the study will have important implications for the scientific understanding of and clinical work with cognitive impairment in MD and PSD. Therefore, we argue that the benefits of the study outweigh the potential risks.

### Trial status and dissemination

Recruitment commenced in October 2022 and is planned to be completed in December 2024. Thirty-five participants were included in the trial as of September 2023. The project will result in three articles published in peer-reviewed international scientific journals. The first article will report primary and secondary outcome findings, i.e. the effect of the intervention on cognition and functional capacity. The second article will investigate baseline predictors of efficacy of the VR-based cognitive remediation intervention. The final article will focus on changes in neuronal activity following the intervention. All results will be published whether negative, inconclusive, or confirming the project hypotheses. Author eligibility will be assessed using the Vancouver Convention, and there will be no utilization of professional writers. The current trials protocol is version 1 dated September 28, 2023.

### Supplementary Information

**Additional file 1:** **Appendix A.** SPIRIT 2013 Checklist: Recommended items to address in a clinical trial protocol and related documents.

**Additional file 2:** **Appendix B.** Supplementary Materials.

## Data Availability

The data sets used and analysed in the current study are available from the corresponding author upon reasonable request.

## Abbreviations

ABCR: Action-based cognitive remediation
AMPS: The Assessment of Motor and Process Skills
BNSS: Brief Negative Symptom Scale
CAVIR: Cognition Assessment in Virtual Reality
COBR: Cognitive Complaints in Bipolar Disorder Rating Scale
CODEL: Cognitive Difficulties in Everyday Life questionnaire
CR: Cognitive remediation
DART: Danish Adult Reading Test
DMN: Default mode network
dPFC: Dorsal prefrontal cortex
FAST: Functional Assessment Short Test
FEAT: FMRIB expert analysis tool
fMRI: Functional magnetic resonance imaging
HDRS-17: Hamilton Depression Rating Scale-17
HMD: Head-mounted display
ISBD: International Society for Bipolar Disorder
ITT: Intention to treat
MD: Mood disorders
MPS: Multimodal Presence Scale for virtual reality environments
NEAD: Neurocognition and Emotion in Affective Disorders Centre
OPUS: The early intervention clinics for psychotic disorders in the Capital Region of Denmark
OTS: One Touch Stockings of Cambridge
PSD: Psychosis spectrum disorders
PSC: Perceived Competence Scale
PQ: Presence questionnaire
RAVLT: Rey Auditory Verbal Learning Test
RBANS: Repeatable Battery for the Assessment of Neuropsychological Status
RVP: Rapid visual information processing
SAPS: Scale for the assessment of positive symptoms
SCAN: Schedules for Clinical Assessment in Neuropsychiatry
SCIP-D: Screen for Cognitive Impairment in Psychiatry-Danish version
SWM: Spatial working memory
TAU: Treatment as usual
TAM: Technological Acceptance Model questionnaire
TMT-A: Trail Making Test A
TMT-B: Trail Making Test B
UPSA-B: Brief Performance-Based Skills Assessment of the University of California San Diego
VR: Virtual reality
VRSQ: Virtual Reality Simulation Sickness Questionnaire
WAIS-III LNS: Wechsler Adult Intelligence Scale Version III Letter–Number Sequencing
WCST: Wisconsin Card Sorting Task
WHOQOL-BREF: World Health Organization’s Quality-of-Life Assessment
YMRS: Young Mania Rating Scale
