# Supplementary material for: Effect of immersive virtual reality-based cognitive remediation in patients with mood or psychosis spectrum disorders: study protocol for a randomized, controlled, double-blinded trial
Source: Trials. 2024 Jan 24;25:82. doi: 10.1186/s13063-024-07910-7 (PMC10809611; doi:10.1186/s13063-024-07910-7)
Supplement: Supplementary file 2 — Additional file 2: Appendix B. Supplementary Materials. [file 13063_2024_7910_MOESM2_ESM.docx]

**Appendix C: Supplementary methods**

*Supplementary methods: Subtasks in the Cognition Assessment in Virtual Reality (CAVIR) test*

Verbal learning is assessed in task 1, in which participants are shown a list with ingredients and instructed to memorize these and take them out of the fridge. Performance is measured by the number of correct ingredients remembered (score range: 0-15). Executive functions are assessed in task 2, in which participants are required to plan and select the order in which to perform different sub-tasks involved in cooking a meal to finish before their guests’ arrival. Here, performance is measured the number of correctly placed tasks that ensure timely completion of the tasks (score range: 0-11). Processing speed is assessed in task 3, in which participants place as many correct ingredients as possible in a pot within 90 seconds based on a key of symbols matching every ingredient (no score range). Working memory is assessed in task 4, in which participants observe and memorize the location of cutlery and flatware in the kitchen cupboards and drawers. Performance is measured by the number of drawers opened until all cutlery and flatware is found (lower indicating better performance). Finally, sustained attention is measured in task 5, in which participants are required to repeatedly check the food in the oven or regulate the oven temperature in response to a specific combination of visual and auditive cues while ignoring irrelevant stimuli. Performance is measured by the number of correct responses (score range: 0-30).

*Supplementary methods: Scoring of The Assessement of Motor and Processing (AMPS)*

Scoring of the AMPS assessment is conducted using the available software Occupational Therapy Assessment Package (OTAP) reporting software. OTAP is based on a many-faceted Rasch measurement model making it possible to convert ordinal raw scores into overall linear ADL motor and process ability measures adjusted for severity of the task, skill item difficulty, and rater severity (determined based on rater calibration).
